# Stress dynamically regulates co-expression networks of glucocorticoid receptor-dependent MDD and SCZ risk genes

**DOI:** 10.1038/s41398-019-0373-1

**Published:** 2019-01-29

**Authors:** Christoph A. Zimmermann, Janine Arloth, Sara Santarelli, Anne Löschner, Peter Weber, Mathias V. Schmidt, Dietmar Spengler, Elisabeth B. Binder

**Affiliations:** 1 Department of Translational Research in Psychiatry, Max Planck Institute of Psychiatry, Munich, Germany; 2 Department of Stress Neurobiology and Neurogenetics, Max Planck Institute of Psychiatry, Munich, Germany; 3 Department of Psychiatry and Behavioral Sciences, Emory University School of Medicine, Atlanta, Georgia

## Abstract

Early-life adversity is an important risk factor for major depressive disorder (MDD) and schizophrenia (SCZ) that interacts with genetic factors to confer disease risk through mechanisms that are still insufficiently understood. One downstream effect of early-life adversity is the activation of glucocorticoid receptor (GR)-dependent gene networks that drive acute and long-term adaptive behavioral and cellular responses to stress. We have previously shown that genetic variants that moderate GR-induced gene transcription (GR-response eSNPs) are significantly enriched among risk variants from genome-wide association studies (GWASs) for MDD and SCZ. Here, we show that the 63 transcripts regulated by these disease-associated functional genetic variants form a tight glucocorticoid-responsive co-expression network (termed GCN). We hypothesized that changes in the correlation structure of this GCN may contribute to early-life adversity-associated disease risk. Therefore, we analyzed the effects of different qualities of social support and stress throughout life on GCN formation across distinct brain regions using a translational mouse model. We observed that different qualities of social experience substantially affect GCN structure in a highly brain region-specific manner. GCN changes were predominantly found in two functionally interconnected regions, the ventral hippocampus and the hypothalamus, two brain regions previously shown to be of relevance for the stress response, as well as psychiatric disorders. Overall, our results support the hypothesis that a subset of genetic variants may contribute to risk for MDD and SCZ by altering circuit-level effects of early and adult social experiences on GCN formation and structure.

## Introduction

Social experiences shape brain structure and function by inducing plastic changes from the early prenatal period until the end of life^1,2^. Above all, early-life experiences, such as maternal stress during pregnancy and child abuse but also supportive parenting, can cause long-lasting changes in neural circuit function and stress hormone regulation that may moderate risk for major depressive disorder (MDD) and schizophrenia (SCZ)^3,4^. Importantly though, the impact of early-life adversity on disease risk is moderated by genetic variation^5^ and later life experiences through biological mechanisms, which are insufficiently understood at present.

Adverse early experiences typically activate the stress hormone system leading to increased glucocorticoid secretion and the activation of glucocorticoid receptors (GRs) by cortisol^6^. GRs reside in the cytoplasm and translocate into the nucleus upon cortisol binding to regulate gene expression through sequence-specific DNA binding or protein interactions with other DNA-bound transcriptional regulators^7^. GR-regulated gene networks coordinate acute and long-term adaptive responses to stress, as well as timely termination of the stress response once the threat has been mastered. Failure to turn-on and -off GR responses efficiently has been proposed to result in “wear and tear” of the body and the brain and facilitates the development of associated pathologies^8^.

Studies on the genetics of gene expression provide a unique opportunity to link DNA sequence variation to phenotypes and disease^9^. We previously hypothesized that genetic variation in GR-regulated gene networks could contribute to the risk for psychiatric diseases^10^. Therefore, we measured glucocorticoid-induced changes in gene expression in genetically diverse individuals to infer GR expression quantitative trait loci (eQTLs). Genetic variants significantly associated with differential GR-induced expression preferentially mapped to long-range enhancer elements consistent with GR’s major mode of transcriptional regulation^11^. Furthermore, these GR-response eQTLs were significantly enriched in brain-specific enhancers and among genetic variants identified in genome-wide association studies (GWASs) for MDD and SCZ^10^. In additional independent samples, these variants also predicted major depression and amygdala (AMY) reactivity to threat in a cumulative manner.

Focusing on MDD risk genes in our initial analyses, we showed that the transcripts moderated by these variants are expressed in the brain, are regulated by stress, and form a tight co-expression network that comprised basic biological processes such as ubiquitination, proteasome degradation, and inflammation. We hypothesized that the cumulative genetic scores for these functional disease variants could change the response of the whole co-expression network to different qualities of social experience and by this influence disease risk. A first step towards testing this hypothesis would be to assess whether indeed exposure to different qualities of social experience can alter the co-expression network structure of these transcripts. This can be tested in animal models exposed to different qualities of social support and stress throughout development and beyond for which the expression levels of the transcripts within these gene networks is measured in different brain regions.

The main intent of our experiment was to explore the dynamics of this glucocorticoid-responsive co-expression network (GCN) (capturing the information from GWAS risk variants) in response to extreme differences in social experiences during early and or adult life, modeling epidemiological risk and protective factors for MDD/SCZ. We have chosen an animal model that contrasts different qualities of social support and stress during different developmental stages to explicitly capture the complexity of social life trajectories^12^.

## Materials and methods

### Genes of interest and network analysis

For this study, 63 genes (see supplementary table 1) that were previously identified as GR-response eQTL and colocalize a risk variant for MDD and/or SCZ as an eSNP were analyzed^10^. A co-expression network of those genes was predicted by GeneMANIA^13^ without the addition of related genes and attributes. To estimate the null distribution, we calculated the gene network for 10 same size sets of randomly chosen GR-response transcripts (*n* = 4383 identified in Arloth et al.^10^). Hub genes were detected by calculating the node degree distribution using the Network Analyzer tool within Cytoscape 3.5.1^14^. The functional annotation was performed using Enrichr (http://amp.pharm.mssm.edu/Enrichr/enrich)^15,16^ focusing on KEGG 2016^17^ and Wikipathways 2016^18^ for pathway enrichment and the gene ontology (GO) terms molecular function, biological processes, and cellular component, all in the version 2017b. The significance tests for these analyses are described in detail in^16^. Briefly, Enrichr implements three approaches to compute an enrichment: (1) Fisher’s exact test, (2) permutation-based Fisher's exact test (generates multiple random gene lists and computes a *z*-score for deviation from the expected rank), and (3) a combined test ((*p*-value of 1) × (*z*-score of 2)). We report the permutation-based *p*-value (referred to as Enrichr *p*-value).

### Animal model

**Fig. 1:**
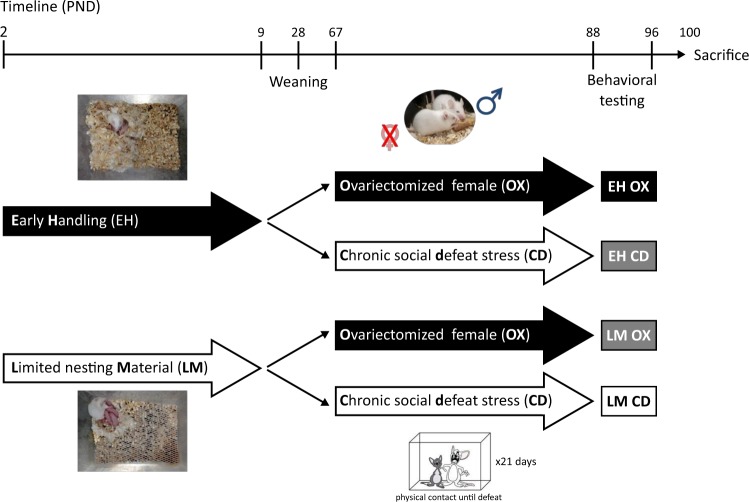
Experimental timeline and social stress conditions. From postnatal day (PND) 2 to PND 9 individual mice litters were randomly assigned to two groups that were exposed to either increased maternal care (defined as early handled (EH)) or fragmented maternal care (limited nesting and bedding material (LM)). On PND 9, all pups returned to standard rearing conditions, were weaned on PND 23, and housed in groups (4 animals/cage). Upon reaching adulthood (PND 67) male mice (EH or LM) were further separated in two groups that were exposed either to a supportive environment (housing with an ovariectomized female; OX) or an aversive environment (chronic social defeat; CD). Collectively, animals experienced either successive supportive or aversive social environments (EHOX or LMCD, respectively) or contrasting social environments (EHCD and LMOX). Theses manipulations were not followed by changes in baseline corticosterone nor significant changes in NR3C1 mRNA expression (as measured by targeted sequencing) encoding the GR. For further details, see^26^

Male Balb/c mice were exposed to either early-life adversity (limited nesting and bedding material; LM) or a caring environment (early handling, EH). LM was performed by placing the animals from postnatal day (P) 2 to P9 in a cage with a metal grid instead of bedding material and reduced nesting material, as described previously^19^, leading to fragmented maternal care. EH was performed by removing the offspring from the maternal cage to a new cage for 15 min per day on P2 to P9, a procedure that has been shown to increase maternal caregiving behavior^20^. A group with an unmanipulated environment (i.e., animal facility reared mice) was explicitly omitted to test only two opposing rearing environments. At adulthood (12 weeks of age), animals of each group were then either housed with an ovariectomized female (OX; supportive social environment) or underwent chronic social defeat stress (CD; aversive social environment) for three consecutive weeks giving rise to four experimental groups exposed to different qualities of social experience (EHOX, EHCD, LMOX, and LMCD) (Fig. 1). See Santarelli et al.^19^ for more detail on physiological and behavioral alterations observed in these groups. We note that theses manipulations were not followed by changes in baseline corticosterone nor significant changes in *NR3C1* mRNA (as measured by targeted sequencing) encoding the GR as described in^19^. From each treatment group, eight mouse brains were analyzed. The animals were sacrificed under basal conditions at 2 h after lights on during the circadian nadir on P100, 12 days after the end of the adult manipulations. Micropunches from desired brain regions were collected under histological control by Cresyl Violet staining according to the Mouse Brain Atlas^21^ and immediately stored at −80 °C. Twelve different brain regions including AMY, bed nucleus of the stria terminalis, cerebellum (CER), dorsal hippocampal Cornu Ammonis (CA) 1 region (dCA1), dorsal hippocampal CA3 region (dCA3), dorsal dentate gyrus (dDG), prefrontal cortex, nucleus accumbens (Nac), paraventricular nucleus (PVN), ventral hippocampal CA1 region (vCA1), ventral hippocampal CA3 region (vCA3), and ventral dentate gyrus were collected (see supplementary Figure 1). These brain regions were selected for their contribution to the regulation of the stress response, as well as their role as targets of stress hormones.

All procedures on animals were approved by the Government of Upper Bavaria and were in conformity with European Union Directive 2010/63/EU. Standard laboratory animal housing conditions were maintained throughout the experiments (unless otherwise stated) and 12-h daily illumination (lights on at 06:00 a.m.).

### RNA extraction

Frozen micropunches were lysed quickly in 500 µl Trizol (QIAzol) reagent and homogenized by repeated passing through an insulin syringe (29G). Subsequently, samples were kept at room temperature for at least 3 min before adding 100 µl chloroform. The remaining steps were conducted according to the miRNeasy Mini Kit (Qiagen, Hilden, Germany) protocol. Elution was performed with 30 µl RNAse-free water with a second round using the eluate from the first round.

### Next-generation sequencing/TruSeq® Targeted RNA sequencing

TruSeq® Targeted RNA sequencing (Illumina, San Diego, USA) was carried out according to the manufacturer’s protocol.

#### Assay design

As a first step, mouse orthologous of GR-response MDD- and/or SCZ-related eQTL genes were mapped (mouse orthologues *n* = 55; see Supp. Table 1) and specific assays for the genes of interest were chosen in the Illumina DesignStudio. Specific primers for these assays were combined by the manufacturer in a targeted oligo pool (TOP). The TOP was applied to the reverse transcribed RNA per protocol. Gene-specific sequences of 50 bp were amplified and supplied with sample-specific indexes to enable pooling during sequencing.

#### Randomization

All samples were randomized for experimental group and brain region during RNA extraction, as well as library preparation to prevent technical batch effects. Libraries were performed according to the manufacturer’s protocol based on the Illumina TruSeq Targeted RNA Expression Kits. We used eight samples per group from our four experimental groups (EHOX, EHCD, LMOX, and LMCD) from 12 brain regions, which summed up to a total number of 384 samples. During RNA extraction, 24 samples were extracted at once, whereas the library preparation was performed in four 96-well plates. Each 96-well plate contained three brain regions, which were distributed randomly into the columns of the plate. Each plate column contained two samples each from the four experimental groups randomly distributed over the rows. Three columns of a 96-well plate were assigned to one RNA extraction from each of the three brain regions on that plate.

After sequencing with the MiSeq Sequencer (V3 chemistry for 150 cycles), sequencing quality was analyzed with FastQC^22^. Low-quality reads with phred scores lower than 20 or a length smaller than 25 bp were removed using PRINSEQ (PReprocessing and INformation of SEQuence data) lite Version 0.20.4^23^. Subsequently, the reads were aligned with Burrows-Wheeler Aligner (BWA) Version 0.7.12^24^ with default settings (allowing a maximum of three mismatches) and the manifest file provided by Illumina as reference.

Next, the gene expression quantification was inferred from the alignment. Genes with low overall coverage, i.e. <2000 reads across all samples, were excluded from further analysis (*n* = 5). The data were then normalized using DESeq2^25^. Finally, surrogate variable analysis (SVA) from the R/Bioconductor package sva^26^ was applied to identify hidden technical batches. To identify outliers, a linear model (LM) was calculated with the normalized expression counts and the five significant SVs (according to Leek et al.^26^). Subsequently, the residuals were clustered and samples more than four standard deviations away from the mean in either of the first two principal components were excluded (*n* = 2 samples). Gene expression values of 50 genes in 382 samples passed the preprocessing.

### Differential expression

The differential expression analysis of the normalized sequencing data were performed in R version 3.2.3 (2015-12-10)^27^. We first tested the distribution of the data per gene and brain region, and—depending on the outcome—subsequently used a generalized linear model with negative binomial distribution or a linear model. All models included the detected significant variables as covariates.

Significance is reported after controlling for multiple testing with a false discovery rate (FDR) lower than 10% for all detectable transcripts within each brain region and across the four conditions and FDR-corrected significance is reported as *q*-values in the results and supplemental table 3.

For analysis of GCN gene expression in human hippocampus, we used the data described in Kang et al.^28^ and deposited as GSE25219. Of the 63 genes within the tested GCN, 40 had data within GSE25219. Dimension reduction using principal component analysis (PCA) was performed on the expression levels of these 40 genes and the first principal component (PC1) was extracted. PC1 was then associated with developmental stages using a linear regression model.

### Correlation network generation

Using the residuals of the normalized and batch corrected gene expression values, the pairwise correlation coefficients between all gene pairs were calculated and adjusted for the expression levels of all other genes using Gaussian Graphical Model implemented in the R package GeneNet.

Partial correlation calculation was performed separately for each brain region (*n* = 12) and condition (*n* = 4) and controlled for the expression levels of all other transcripts, resulting in 48 networks. Partial correlation coefficients are not directly comparable to Pearson's correlation coefficients as they control for confounding effects of all other transcripts. The pairwise partial correlation between transcripts A and B will be calculated correlating A – (Z_1_ to Z_n_) and B – (Z_1_ to Z_n_), so that partial correlation coefficient will be numerically lower than Pearson's correlation coefficients. Statistical significance of the partial correlations was assessed using the empirical Bayes local FDR statistic^29^ and partial correlations at an FDR < 0.2 were considered as significant according to Schäfer et al.^30^. Networks were visualized with the R Bioconductor package Rgraphviz, where each gene corresponds to a node and edges represent the dependencies, i.e., partial correlation coefficient, between them. The network properties, i.e., number of edges, number of nodes, absolute sum of partial correlation coefficients, and link density were analyzed using iGraph and visualized in pheatmaps. Edges were defined as pairwise partial correlations with an FDR < 0.2 and node degree as the number of such significant edges connected to a node. A node was defined as a transcript with at least one edge.

## Results

### Network analysis of GR-response eQTL genes related to MDD and SCZ

**Fig. 2:**
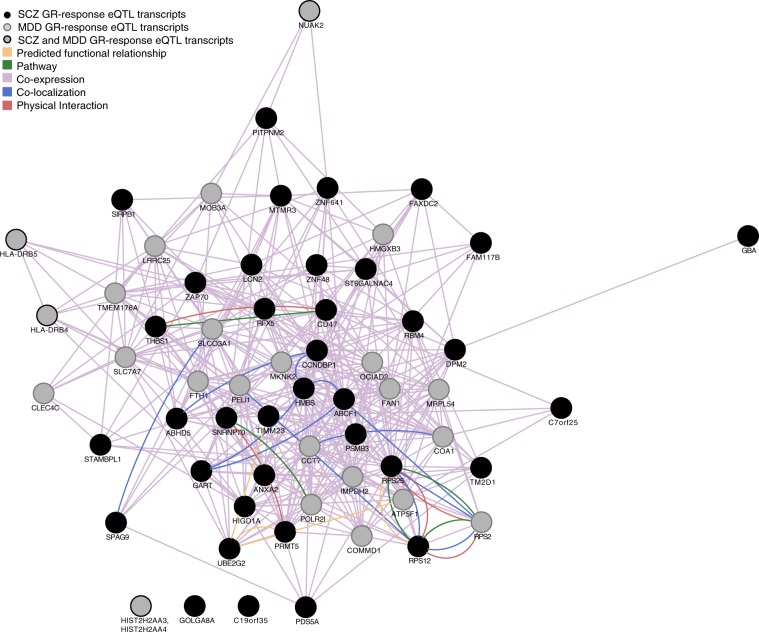
Human GeneMANIA gene network. The glucocorticoid receptor (GR)-dependent expression quantitative trait loci (eQTL) network comprises risk genes for major depressive disorder (MDD; gray filled circles, *n* = 20), schizophrenia (SCZ; black filled circles, *n* = 39) and both (gray circles with black border, *n* = 4). It forms a tightly interconnected gene network termed GCN. The edge colors indicate the type of interaction, as explained in the legend on the top left. Information on individual risk genes is provided in Supplemental Table 1

In our previous publication^10^, we have analyzed the human network properties of the GR-eQTL transcripts related to MDD risk only (*n* = 24 transcripts). Given the additional significant enrichment of GR-eQTLs among schizophrenia risk loci and the significant SNP-based co-heritability^31^ between these two disorders, we now investigated if the 63 GR-response eQTL transcripts (see Supplementary Table 1 for details and abbreviation of gene names) for which the regulating SNPs are nominally associated with MDD (*n* = 20) or SCZ (*n* = 39) or both (*n* = 4), form a common transcription network structure. GeneMANIA was used to generate such a GR-eQTL/GWAS gene network (GCN, see Fig. 2) using the full GeneMANIA database including data from multiple tissues. The GCN included 61 transcripts of the 63 transcripts (*AI655567* and *RPL23AP64* were not included in the GeneMANIA database and both are regulated by SNPs associated with SCZ). Of these, 58 transcripts formed a tight network with 423 edges. Only three transcripts were isolated nodes (see Fig. 2). Within this network the category direct “physical interactions” (i.e., protein–protein interactions) was the most enriched type of interaction (fold enrichment of 1.63) over random networks. Testing for pathway (KEGG 2016 and Wikipathways 2016) and GO term enrichment of the 58 genes within the network, we observed significant enrichment for GO cellular components 2017b, namely the terms cytoplasmic side of late endosome membrane, lumenal side of late endosome membrane and multivesicular body membrane, see Supplemental Table 4. For WikiPathways, cytoplasmic ribosomal proteins and inflammatory response pathway were found to be significantly enriched, see Supplemental Table 5.

In order to identify the most connected genes, the node degree of each gene—i.e., the number of significant edges of this node—was calculated. *RPS25* (ribosomal protein S25) was the top connected gene with a node degree of 30. This gene was followed by *CCNDBP1* (cyclin D1 binding protein 1) (28 nodes) and *HMBS* (hydroxymethylbilane synthase) (27 nodes) (see Supplemental Table 2 for more detail on the network).

These data provide support that GR-response eQTL transcripts with eSNPs associated with MDD and/or SCZ form a tightly connected network.

### Developmental trajectory of GCN gene expression in human hippocampus

Using hippocampal gene expression data from embryonic to adult postmortem brains of the Human Brain Transcriptome atlas^28^ in which 40 of our 63 risk genes were expressed, we observed that first principal component of the expression levels of these 40 genes is significantly associated with developmental stage, *p* = 1.4e–36. The developmental trajectories of each of these genes in depicted in Supplemental Figure 2. This supports that the large majority of these transcripts are expressed in human hippocampus and regulated across brain development.

### Differential expression analysis of GR-response MDD- and SCZ-related eQTL risk genes in various brain regions

To establish whether the GCN transcripts are regulated by different qualities of social experience, including adverse, stressful experiences that are established risk factors for both psychiatric disorders, we used a mouse model^19^ that combined both early stress or supportive exposures with either adult stress or supportive exposures (see Fig. 1). We first investigated whether the orthologues of the 61 GCN transcripts were differently regulated across the 12 different brain regions for each of the four experimental conditions (early and adult support: EHOX, early support and adult stress: EHCD, early stress and adult support: LMOX, and early and adult stress: LMCD). Of the 61 transcripts, 55 had a mouse orthologue. Of these 55, 50 transcripts showed expression levels that were above detection threshold and were thus included in the analysis.

The univariate analyses are summarized for each brain region in Supplemental Table 3. In univariate analyses of condition/brain region, early-life stress led to a significant change of Snrnp70 gene expression in Nac with a *q*-value of 0.016. Exposure to chronic social defeat stress in adulthood resulted in significant changes of seven genes in four different brain regions (dCA1: *Fth1, Mrpl54, Abcf1, Psmb3, Rbm4*; dDG: *Hist1h2al*; Nac: *AW209491*; vCA1: *Adhd5*; see Supplementary Table 3). The human orthologues of the differentially expressed genes in mice *MRLP54*, *ABCF1*, *PSMB3*, *FTH1*, and *ADHD5* had relatively high connectivity (degree (*d*) = 17–25), in the above-described human GCN network, whereas *C7orf25* (orthologue of *AW209491*) had a very low degree of 4 and *HIST2H2AA3, d* = *0*, falls on an edge (see Fig. 2). The interaction of early-life adversity and adult chronic stress on differential expression showed significant effects of five genes in four different brain regions (AMY: *Higd1a*, dCA1: *Ccndbp1*, dDG: *Rsp12, Rsp25* and Nac: *Adhd5*; see Supplementary Table 3). Interestingly, two of these genes are major human GCN genes with a high node degree including the hub genes *RSP25* (*d* = 30), *CCNDPD1* (*d* = 28), as well as *ADHD5* (*d* = 18) and *HIGD1A* (*d* = 19).

Taking into account additional tests related to the number of brain regions, only the effect of CD on *C7orf25* (orthologue of *AW209491*) in the Nac would remain significant after correction for multiple testing. Collectively, these data thus indicate that robust differential expression of single GCN risk genes with large effects is a rare event in response to different social stress experiences.

### Brain region-specific GCN formation in different stress condition

**Fig. 3:**
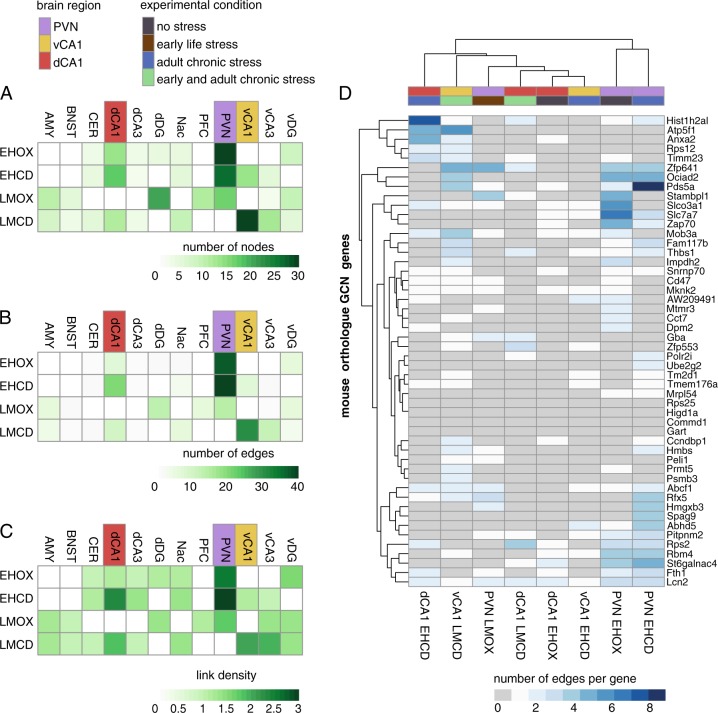
Stress-dependent and tissue-specific GCN formation and their properties. Heatmaps show the total number of edges (**a**) and nodes (**b**) for each GCN, as well as the link density (**c**) in the respective GCNs. Overall, GCN formation is highly responsive to different stress histories and tissue-specific. (**d**) Heatmap in terms of the number of edges per GCN gene in the hypothalamic paraventricular nucleus (PVN) and hippocampal dCA1 and vCA1 regions

Given the absence of strong single gene effects, we next performed a comprehensive co-expression analysis of the 50 orthologue GCN genes across the 12 different brain regions and the four conditions. All pairwise correlations were adjusted for the expression levels of all other genes in the GCN, by calculating partial correlation. Figure 3 summarizes all investigated network parameters (number of edges, number of nodes, the partial correlation coefficient, and link density). We detected major differences in network strength dependent both on the previous stress history and the specific brain region. Mainly, in the PVN, a central driver of the stress response, we observed a strong (number of nodes *n* = 30 vs. mean = 5.9), well-structured (number of edges *n* = 38 vs. mean = 5.3), and dense (link density *n* = 2.53 vs. mean = 0.8) GCN in the absence of any stress history (EHOX). The co-expression network in this brain region was completely absent in mice exposed to both, early and late social stress (LMCD, all measures = 0) and to a lesser degree in mice exposed to early adversity only (LMOX, nodes *n* = 17 vs. mean = 6.7, edges *n* = 15 vs. mean = 4.6, link density *n* = 1.76 vs. mean = 0.8). On the other hand, chronic adult stress without a history of early-life adversity (EHCD, nodes *n* = 27 vs. mean = 6.6, edges *n* = 40 vs. mean = 8.6, link density *n* = 2.96 vs. mean = 0.9) did not alter paraventricular network formation when compared with stress-free mice (EHOX). This suggests stronger effects of early adversity on the GCN in PVN than adult chronic stress, as well as a cumulative effect of stress exposure on the strength of this network.

The brain region with the second largest dynamic network changes across the four conditions was the vCA1. In contrast to the PVN, the vCA1 region, sharing a major role in stress regulation^32^ did not express the GCN (all network parameters 0) in the absence of any stress history (EHOX), but GCN network strength emerged with exposure to adult stress (EHCD, nodes *n* = 14, edges *n* = 8, link density *n* = 1.14) and was further strengthened by combined exposure to early and adults stress (LMCD, nodes *n* = 30, edges *n* = 31, link density *n* = 2.07). However, exposure to early-life adversity alone (LMOX) was not associated with network formation.

It is important to note that changes in GCN formation were not confined to the PVN and hippocampal vCA1 region, but observed also in other brain regions although to a much lower degree (see Fig. 3). These findings suggest that GCN formation is moderated by different qualities of social environment in a brain region-specific manner in circuits central to stress regulation such as those including the PVN and the vCA1.

### Brain region-specific GCN formation in different stress condition

Brain region-specific social environment-responsivity raises the question whether the network properties of the underlying risk genes are preserved across brain regions and conditions. Therefore, we next investigated the number of edges per GCN gene in the PVN and the vCA1 in those conditions exhibiting GCN formation (PVN: EHOX, EHCD, LMOX and vCA1: EHCD, LMCD), and also included data from the dCA1 (EHOX, EHCD, LMCD), the third most connected region (although with quite a distance (see Fig. 3b)) for comparison. Hierarchical clustering analysis (see Fig. 3d) revealed that the two GCNs in the PVN under the low early stress conditions cluster closely together, suggesting that adult chronic stress does not strongly impact the edge structure of the PVN network (i.e., similar connectedness of the network genes), whereas it is disrupted with early stress exposure. A different picture emerged in the vCA1, were the two conditions with the highest overall connectedness of the GCN (EHCD and LMCD) showed dissimilar edge properties and were not the next neighbors in the clustering analysis.

**Fig. 4:**
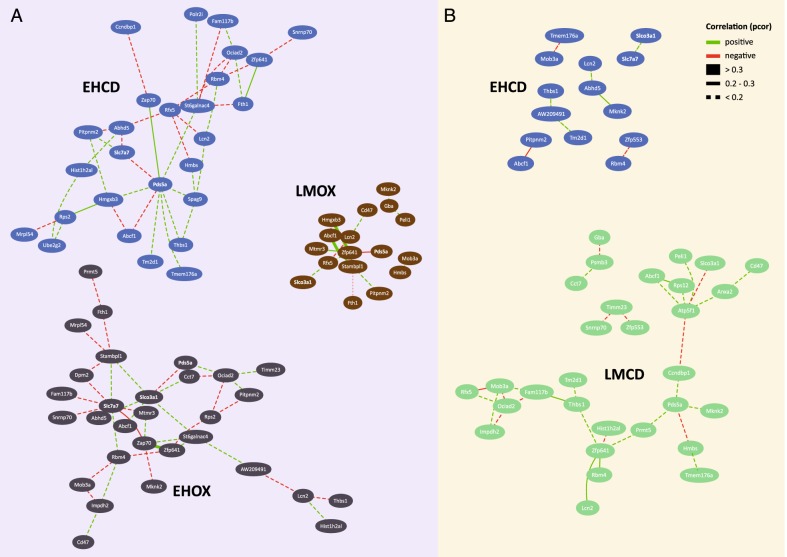
Paraventricular and hippocampal GCN topology in stress-treated mice. Paraventricular (**a**) and ventral CA1 (**b**) GCNs of mice that successively experienced different kinds of supportive or aversive environments in early (EH vs LM) or adult (OX vs CD) life are shown. Negative or positive partial correlations are marked in red or green, respectively, with partial correlation strengths represented by the edge style

We then explored the exact topology of the stress-dependent GCNs in the PVN and the vCA1 (see Fig. 4). We first noted that both, MDD and SCZ-associated transcripts contributed proportionally (38% MDD genes in original selection, range of MDD genes in networks 29–35%) to each of the networks, indicating the absence of disease-specific involvement in any of the regulated GCN networks. When focusing on the PVN, we noted that the network under conditions of social support (EHOX) contained 30 of the 50 orthologue transcripts and that eight of the network genes had more than four connections. In the adult stress only condition (EHCD), a network of similar strength was observed (27 transcripts) sharing more than half (*n* = 16) transcripts with the EHOX network. Interestingly, six of the eight genes with more than four connections in the EHOX network were also included in the EHCD network, namely *Slc7a7, Rbm4, Zap70, St6galnac4, Zfp641*, and *Ociad2* and the majority of them (*n* = 4), again had four or more connections. This suggests that while these two PVN networks are not identical, they share a number of key features, supporting that adult stress also does not have a major impact on the network topology. However, one has to note that the centrality and connectivity pattern of some transcripts were affected by the additional stress condition. For example, Psd5a with two edges in the EHOX condition, moved to a more central position in the EHCD condition, with nine edges. As described above, this paraventricular co-expression network is disrupted with exposure to early adversity, especially when followed by adult chronic stress.

Contrary to the PVN, the vCA1 GCN was mainly apparent in the combined stress condition (LMCD). Here 30 of the 50 transcripts formed a correlated expression network, with 16 genes shared with low stress PVN network (EHOX) and an additional six, which were only present in the adult only stress (EHLM) PVN network. Of the eight more highly connected genes in the PVN EHOX condition, four were also included in the vCA1 LMCD network, however, only two of them still showed a high connectivity (*Ociad2* and *Zfp641*). This suggests that while a substantial number of transcripts are shared between the low early stress PVN network and the combined stress vCA1 network, the topology of this network differed more than between the two PVN networks. Interestingly, *Psd5*—the most connected gene in the PVN adult stress network (eight edges) was also part of the vCA1 combined stress network and showed four connections, suggesting that connectivity of this gene may more specifically relate to chronic adult stress.

## Discussion

In this article, we report that 63 transcripts for which GR-induced gene expression was modulated by MDD/SCZ-associated SNPs form a tightly interconnected gene expression network (GCN). This raises the question whether this GCN could respond to differences in social experiences, especially early-life stress, an important risk factor for MDD and SCZ. When analyzing the effects of different qualities of social stress in contrast to different qualities of social support on GCN formation across distinct brain regions during distinct developmental phases in mice, we observed that these different social experiences substantially affect GCN formation and structure. These changes were highly brain region-specific and apparent mainly in two functionally interconnected regions, the ventral hippocampus and the hypothalamus (see below). The fact that the transcripts within this network are highly regulated across human hippocampal development suggests a role in neuronal development and their developmental trajectory may serve as a substrate for early-life experiences to influence differential GCN formation.

When annotating the 58 transcripts connected within this GCN, we found enrichment of the GO cellular components “late endosome membrane” and “multivesicular membrane”, both contributing to endocytosis. Regulation of endosomal traffic is a critical component of synaptic growth and development. Many of the synaptic growth mutants identified in *Drosophila* alter endocytic trafficking: mutations disrupting the formation of signaling endosomes cause reduced synaptic growth, while mutations altering traffic to the recycling endosome or lysosome cause synaptic overgrowth due to enhanced signaling^33^. Endosomal regulation of signaling pathways in synaptic growth and development appears critical for activity-dependent circuit refinement and are proposed to contribute to risk for MDD and SCZ^34,35^. In support of this hypothesis, postmortem brain studies showed these pathways to be altered in both SCZ and MDD^36^. This suggests that alterations in stress-induced changes of endocytosis could contribute to disease risk by altering neuronal activity. For example, glutamate receptor turnover has been shown to be altered by glucocorticoid-induced enhancement of ubiquitin/proteasome-mediated degradation of receptor subunits^37^, a process localized to late endosomes and multivesicular membranes^38,39^. Pathway analyses revealed enrichment for cytoplasmic ribosomal proteins; ribosomal protein have been shown to be altered both in postmortem gene expression studies for MDD and SCZ^36^, as well as in pluripotent stem cell-derived neural progenitor cells^40^ or olfactory-derived neuroepithelial cells^41^ from patients with SCZ.

The second enriched pathway was inflammation, a system not only strongly implicated in both disorders by a large body of experimental evidence^42,43^ but also from recent cross-disorder annotations of GWASs including both disorders^44^.

Furthermore, proteins relevant to the pathways enriched in the total network described above are also encoded by transcripts that are part of the dynamically regulated networks in both the PNV and the ventral CA1. These include *Snrnp70, Rps2, Rps12, Mrpl54, Rbm4* for ribosomal processes; *Zap70* and *Rfx5* for immune-related functions, and Anx2 for endocytosis (Fig. 4), suggesting the experience-specific co-regulation of these pathways.

Finally, transcripts with eSNPs associated with either MDD or SCZ contributed to a similar degree to these dynamically regulated networks in the animal model. This suggests that genes associated with either disorder may impact networks sensitive to different qualities of social experiences. For the development of human pathology, it raises the question whether one would observe disease-specific changes in GCN with exposure to adversity. One possibility could be that disease-specific genetic variation would impact distinct hubs in the same network leading to similar GCN alterations. For example, in the network emerging with combined stress in the ventral hippocampus, the four “hub” transcripts are influenced by both SCZ-associated variants (*PSD5* and *ZFP641*) and MDD-associated variants (*OCIAD2* and *ATP5F1*) (see Fig. 4). However, additional experiments in human tissue are necessary to test this hypothesis.

By comparing GCNs in the combined support vs. the combined stress condition, we observed opposite GCN formation within the vCA1 region and the PVN, with other regions showing little or no co-expression structure (see Figs. 3a-c). These two functionally highly interconnected regions are critical for a well-organized stress response and stress-related psychiatric disorders^32,45^. Thus, intercorrelated changes in GCN formation could reflect changes in region-specific molecular signatures that evolve in response to social experiences and additionally integrate reciprocal feedback mechanisms between interdependent domains. While we can conclude that different qualities of social experiences alter GCN formation and structure in a brain region specific, and possibly functionally interdependent manner, we cannot as easily extrapolate whether these processes associate with risk or resilience to stress-related phenotypes. In any case, our results are compatible with the hypothesis that MDD and SCZ manifest circuit-level disorders in which several functionally interconnected brain regions are affected^46,47^ and point to a possible role of altered, or even disrupted, GCN formation as potential risk factor for disease.

In fact, recent data from postmortem brain gene expression studies support a role for changes in co-expression network strength in both MDD and SCZ^48,49^. Labonté et al.^49^, for example, observed not only differentially expressed genes with MDD in multiple brain regions, but also significant changes in network strength of specific co-expression modules. The authors reported mainly gain of connectivity in a series of MDD-associated co-expression modules in a sex-specific but most importantly also brain region-specific manner^49^. The changes in postmortem MDD brain were overlapping with changes observed in an animal model of depression, the chronic social defeat paradigm (CD) also used here. The same group had previously reported differential changes of co-expression networks and changes in network strength, suggesting that changes in co-expression strength has the potential to impact cellular function in animals susceptible or resilient to this stress paradigm in a coordinated manner across brain regions, including the ventral hippocampus^50^. In fact, among the 30 genes constituting the network emerging in the ventral CA1 in the combined early and late stress group in our experiments, five genes (*Lnc2*, *Impdh2*, *Anxa2*, *Rps2*, and *Psmb3*) also showed significant changes in gene expression in the ventral hippocampus between resilient and susceptible animals and control animals.

Overall, our data support the hypothesis that genetic risk variants for MDD and SCZ could influence coordinated gene network properties across brain regions in response to different qualities of social experiences. In human studies, differences in social experiences ranging from early adversity to supportive social networks have been associated with differences in risk or resilience to MDD and SCZ. Targeting multifaceted dysregulation of risk gene co-expression networks within interconnected brain regions in MDD and SCZ may offer an interesting entry point for therapy and may lead to more effective treatments than simply modulating single differentially expressed risk genes^51^.

## Supplementary information

Legend for supplementary files

Supplemental Figure 1

Supplemental Figure 2

Supplemental Table 1

Supplemental Table 2

Supplemental Table 3

Supplemental Tables 4 and 5
