# Supplementary material for: Stress dynamically regulates co-expression networks of glucocorticoid receptor-dependent MDD and SCZ risk genes
Source: Transl Psychiatry. 2019 Jan 29;9:41. doi: 10.1038/s41398-019-0373-1 (PMC6351530; doi:10.1038/s41398-019-0373-1)
Supplement: Supplementary file 1 — Legend for supplementary files [file 41398_2019_373_MOESM1_ESM.docx]

**Supplementary Files**

**Figure S1.** Anatomical localization of the 12 punch areas represented on a sagittal cross section of the mouse brain**.** Brain regions selected for tissue extraction are marked with red circles. Hippocampal regions CA1, CA3 and DG are punched separately for ventral and dorsal parts. AON, anterior olfactory nucleus; Apit, anterior pituitary; ARC, arcuate nucleus; BLA, basolateral amygdala; BST, bed nucleus of the stria terminalis; CA1-3, subregion 1-3 of Ammon’s horn; CC, corpus callosum; CeA, central nucleus of the amygdala; Cereb, cerebellum; CingCx, cingulate cortex; DBB, diagonal band of Broca; DG, dentate gyrus; EWcp, centrally projecting Edinger–Westphal nucleus; FrCx, frontal cortex; IC, inferior colliculus; IPit, intermediate pituitary; LC, locus coeruleus; LS, lateral septum; MS, medial septum; NAc, nucleus accumbens; NTS, nucleus tractus solitarii; OB, olfactory bulb; OccCx, occipital cortex; PAG, periaqueductal gray; ParCx, parietal cortex; PPit, posterior pituitary; PVN, paraventricular nucleus; RN, raphe nuclei; SC, superior colliculus; SN, substantia nigra; SON, supraoptic nucleus; VLM, ventrolateral medulla; VMH, ventromedial hypothalamus; VTA, ventral tegmental area.

**Figure S2.** Scatterplots of the GCN genes (n=40) and their hippocampal gene-level expression from post-mortem brains of the Human Brain Transcriptome atlas (GSE25219,([28](#_ENREF_28))) versus developmental stages (N = 82 samples). Colors indicate the development stages with stage 1-4 reflecting samples at 5-13 PCW, 5-7 at 16-37 PCW, 8-9 at 4-12 months and 10-100 >1 year.

**Supplementary table 1**: GR-response eQTL genes associated with major depressive disorder and/or schizophrenia (XLSX)

- Human Gene: GR-response eQTL gene symbol
- GWAS: Name of GWAS
- GWAS p-Value: p-value extracted from GWAS
- SNP: SNP identifier
- Mouse orthologue: mouse ortholog gene of human GR-eQTL gene
- Transcript ID
- Gene Name
- Ensembl
- EntrezGene
- Remark: gene selection information

**Supplementary table 2**: GCN network attributes (XLSX)

- Gene: Gene symbol
- Clustering coefficient: ratio N / M, where N is the number of edges between the neighbors of n, and M is the maximum number of edges that could possibly exist between the neighbors.
- Degree: the node degree of a node n is the number of edges linked to n.

**Supplementary table 3**: Supplementary table 3 includes a tab for each brain region (XLSX): Significantly differentially expressed GCN genes in mice exposed to different qualities of stress. Genes are reported for either a significant effect of early life treatment (EHOX and EHCD vs LMOX and LMCD) or a significant effect of adult life treatment (LMOX and EHOX vs LMCD and EHCD) as well as the significant interaction of both effects. Differential expression GCN genes was assessed in the amygdala (AMY), the bed nucleus of the stria terminalis (BNST), cerebellum (CER), different subfields of the hippocampus (ventral and dorsal CA1, CA3, DG, respectively), the prefrontal cortex (PFC), the nucleus accumbens (NAC), and the hypothalamic paraventricular nucleus (PVN).

- Gene: Gene symbol
- FC_Adult: Fold change of LM vs. EH
- p_Adult: p-values which represent the significance of a regression model for adult life treatment
- FC_ELS: Fold change of OX vs. CD
- p_ELS: p-values which represent the significance of a regression model for early life treatment
- p_Adult.ELS: p-values which represent the significance of a regression model for the interaction effect of early and adult life treatment
- q_Adult: FDR-adjusted p-values (q-vales) which represent the significance of a regression model for adult life treatment
- q_ELS: FDR-adjusted p-values (q-vales) which represent the significance of a regression model for early life treatment
- q_Adults.ELS: FDR-adjusted p-values (q-vales) which represent the significance of a regression model for the interaction effect of early and adult life treatment

**Supplementary table 4** shows the top 5 enriched GO terms from Enrichr analyses of the 58 genes within the GCN network. “Overlap” refers to the number of transcripts from this analysis overlapping with the genes in the pathways. Enrichr p-value refer to the adjusted p-value (see methods). “Genes” lists the overlapping genes.

**Supplementary table 5** shows the top 5 enriched pathways Wiki-pathways from Enrichr analyses of the 58 genes within the GCN network. “Overlap” refers to the number of transcripts from this analysis overlapping with the genes in the pathways. Enrichr p-value refer to the adjusted p-value (see methods). “Genes” lists the overlapping genes.
