# Supplementary figures and images for: Stress dynamically regulates co-expression networks of glucocorticoid receptor-dependent MDD and SCZ risk genes
Source: Transl Psychiatry. 2019 Jan 29;9:41. doi: 10.1038/s41398-019-0373-1 (PMC6351530; doi:10.1038/s41398-019-0373-1)

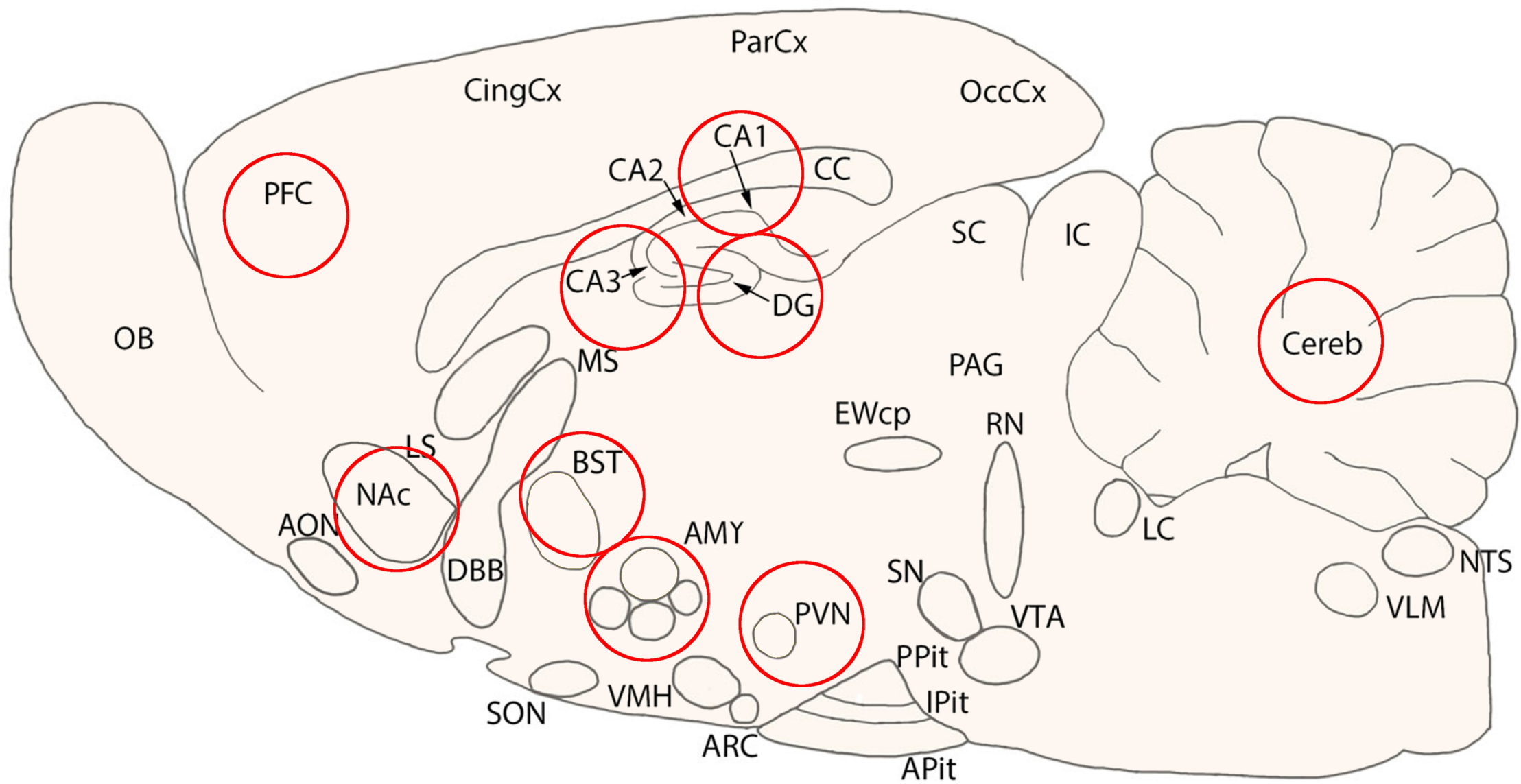

Supplement: Supplementary file 2 — Supplemental Figure 1 [file 41398_2019_373_MOESM2_ESM.pdf]

hippocampal gene-level expression

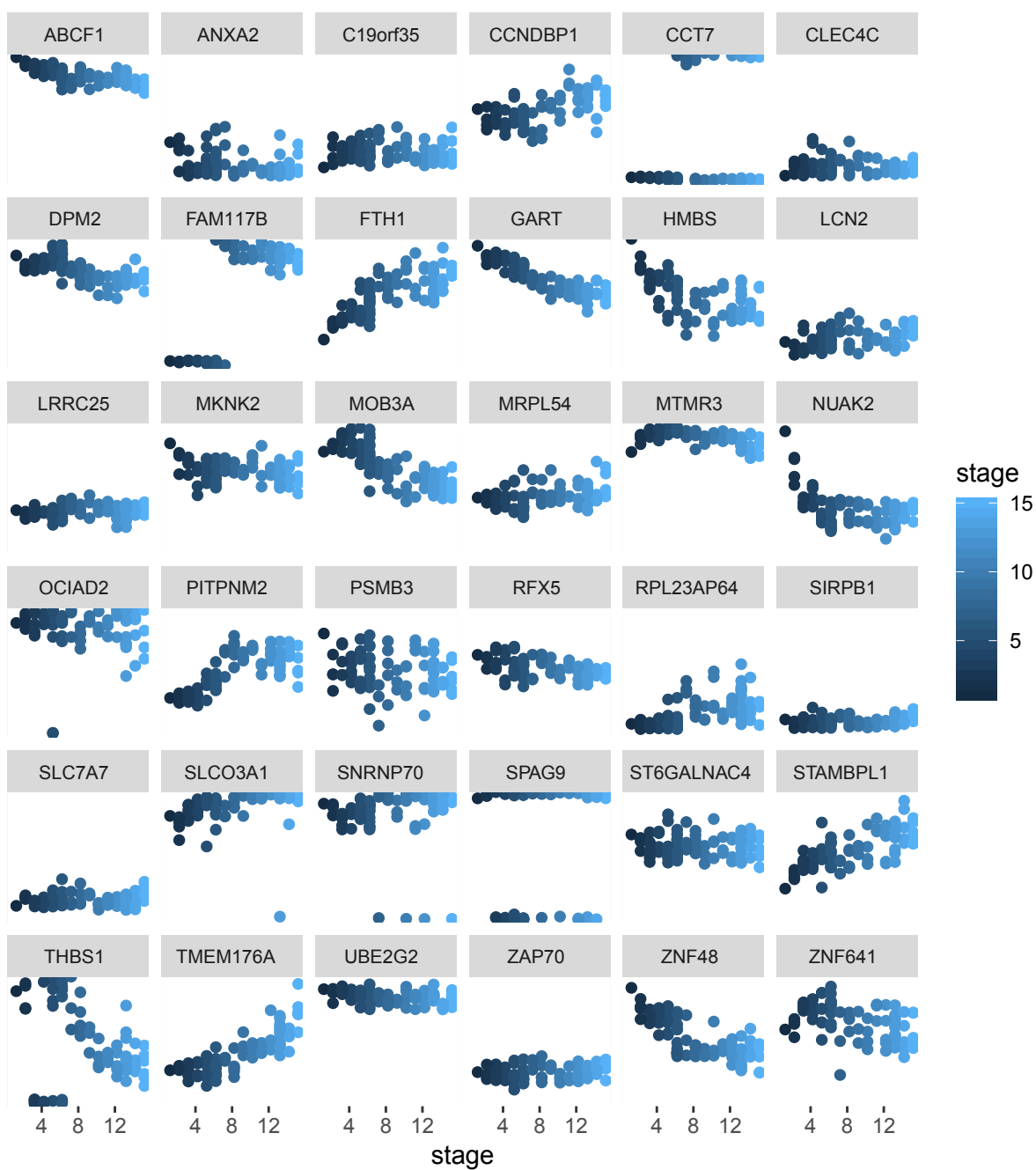

Supplement: Supplementary file 3 — Supplemental Figure 2 [file 41398_2019_373_MOESM3_ESM.pdf]
