# Supplementary material for: Stress dynamically regulates co-expression networks of glucocorticoid receptor-dependent MDD and SCZ risk genes
Source: Transl Psychiatry. 2019 Jan 29;9:41. doi: 10.1038/s41398-019-0373-1 (PMC6351530; doi:10.1038/s41398-019-0373-1)
Supplement: Supplementary file 7 — Supplemental Tables 4 and 5 [file 41398_2019_373_MOESM7_ESM.pdf]

Suppl. Table 4

GO Term cellualr component 2017b	Overlap	Adjusted P-value	Genes
**cytoplasmic side of late endosome membrane (GO:0098560)**	3/39	**0,0226**	HLA-DRB5;HLA-DRB4;ANXA2
**lumenal side of late endosome membrane (GO:0098551)**	3/36	**0,0226**	HLA-DRB5;HLA-DRB4;ANXA2
**multivesicular body membrane (GO:0032585)**	3/53	**0,0375**	HLA-DRB5;HLA-DRB4;ANXA2
lysosomal HOPS complex (GO:1902501)	4/222	0,0765	HLA-DRB5;HLA-DRB4;ANXA2;GBA
integral component of lysosomal membrane (GO:1905103)	4/223	0,0765	HLA-DRB5;HLA-DRB4;ANXA2;GBA
extrinsic component of lysosome membrane (GO:0032419)	4/222	0,0765	HLA-DRB5;HLA-DRB4;ANXA2;GBA
cytoplasmic side of lysosomal membrane (GO:0098574)	4/226	0,0765	HLA-DRB5;HLA-DRB4;ANXA2;GBA
lysosomal proton-transporting V-type ATPase complex (GO:00	4/226	0,0765	HLA-DRB5;HLA-DRB4;ANXA2;GBA
cytolytic granule membrane (GO:0101004)	4/226	0,0765	HLA-DRB5;HLA-DRB4;ANXA2;GBA
endolysosome membrane (GO:0036020)	4/233	0,0765	HLA-DRB5;HLA-DRB4;ANXA2;GBA
phagolysosome membrane (GO:0061474)	4/257	0,1006	HLA-DRB5;HLA-DRB4;ANXA2;GBA
azurophil granule membrane (GO:0035577)	4/273	0,1094	HLA-DRB5;HLA-DRB4;ANXA2;GBA

Suppl. Table 5

Wikipathway 2016	Overlap	Adjusted P-value	Genes
**Cytoplasmic Ribosomal Proteins_Mus musculus_WP163**	3/70	**0,0478**	RPS25;RPS2;RPS12
**Cytoplasmic Ribosomal Proteins_Homo sapiens_WP477**	3/89	**0,0478**	RPS25;RPS2;RPS12
**Inflammatory Response Pathway_Mus musculus_WP458**	2/30	**0,0478**	ZAP70;THBS1
**Inflammatory Response Pathway_Homo sapiens_WP453**	2/30	**0,0478**	ZAP70;THBS1
Purine metabolism_Mus musculus_WP2185	3/158	0,1217	IMPDH2;POLR2I;GART
TGF-beta Signaling Pathway_Homo sapiens_WP366	2/132	0,2550	STAMBPL1;THBS1
